# Molecular Identification of Host Blood Meals and Detection of Blood Parasites in *Culicoides* Latreille (Diptera: Ceratopogonidae) Collected from Phatthalung Province, Southern Thailand

**DOI:** 10.3390/insects13100912

**Published:** 2022-10-08

**Authors:** Sakone Sunantaraporn, Thanaporn Hortiwakul, Kanyarat Kraivichian, Padet Siriyasatien, Narisa Brownell

**Affiliations:** 1 Medical Science Program, Faculty of Medicine, Chulalongkorn University, Bangkok 10330, Thailand; 2 Center of Excellence in Vector Biology and Vector Borne Diseases, Department of Parasitology, Faculty of Medicine, Chulalongkorn University, Bangkok 10330, Thailand; 3 Division of Infectious Diseases, Department of Internal Medicine, Faculty of Medicine, Prince of Songkla University, Songkhla 90110, Thailand; 4 Department of Parasitology, Faculty of Medicine, Chulalongkorn University, Bangkok 10330, Thailand

**Keywords:** biting midges, blood meal, Haemosporidians, Trypanosomatids, Thailand

## Abstract

**Simple Summary:**

Biting midges, which feed on a variety of hosts, transmit a wide range of harmful human and animal viruses and parasites. According to recent studies in Thailand, biting midges may act as potential vectors for leishmaniasis and trypanosomiasis. The purpose of this study is to look for *Leishmania* and *Trypanosoma* DNA in biting midges obtained in the field in a leishmaniasis-endemic area in Phatthalung province, Southern Thailand. In addition, we analysed engorged midges for host blood DNA and screened the collected midges for avian haemosporidian parasites. According to our findings, biting midges have a diversified feeding habit and may be able to transmit various pathogens, including trypanosomatid and avian haemosporidian parasites.

**Abstract:**

Five hundred and fifty-nine female biting midges were collected, and seventeen species in six subgenera (*Avaritia, Haemophoructus, Hoffmania, Meijerehelea, Remmia*, and *Trithecoides*) and two groups (*Clavipalpis* and *Shortti*) were identified. The dominant *Culicoides* species was *C. peregrinus* (30.94%), followed by *C.* subgenus *Trithecoides*. From blood meal analysis of engorged biting midges, they were found to feed on cows, dogs, pigs, and avians. The majority of blood preferences of biting midges (68%; 49/72) displayed a mixed pattern of host blood DNA (cow and avian). The overall non-engorged biting midge field infectivity rate was 1.44 % (7/487). We detected *Leucocytozoon* sp. in three *Culicoides* specimens, one from each species: *C. fulvus*, *C. oxystoma*, and *C.* subgenus *Trithecoides*. *Crithidia* sp. was found in two *C. peregrinus* specimens, and *Trypanosoma* sp. and *P. juxtanucleare* were separately found in two *C. guttifer*. More consideration should be paid to the capacity of biting midges to transmit pathogens such as avian haemosporidian and trypanosomatid parasites. To demonstrate that these biting midges are natural vectors of trypanosomatid parasites, additional research must be conducted with a greater number of biting midges in other endemic regions.

## 1. Introduction

The rising prevalence of vector-borne parasitic infections has raised global health awareness. Some zoonotic infections can be transmitted to humans by blood-sucking vectors that routinely feed on other animals. *Leishmania* and *Haemosporidia* infection are vector-borne parasitic diseases that their potential vectors, *Culicoides* biting midges, also feed on humans [1].

Leishmaniasis is an emerging infectious disease in Thailand, caused by the *Leishmania* parasite, an obligate intracellular protozoon. The main clinical presentations of leishmaniasis can be divided into three forms: visceral leishmaniasis (VL), mucocutaneous leishmaniasis (MCL), and cutaneous leishmaniasis (CL). All cases of leishmaniasis found in Thailand between 1960 and 1999 were brought in by immigrants or returning visitors from endemic areas. The first case of autochthonous leishmaniasis was reported in 1999 [2]. Since then, the reports of autochthonous leishmaniasis cases in humans have been increasing. To date, more than 20 cases of autochthonous leishmaniasis have been confirmed in Thailand. The three major species causing indigenous human leishmaniasis in Thailand include *Leishmania martiniquensis*, *L. orientalis* (previously reported “*L. siamensis*”), and *L. infantum.* Among these three species, the most frequently identified species is *L. martiniquensis* belonging to the subgenus *Mundinia* [3]. The majority of *Leishmania* parasite transmission occurred through the bite of infected female phlebotomine sand flies (Diptera: Psychodidae), except for *Mundinia*. Natural vectors of the recently discovered *Leishmania* subgenus remain inconclusive. Recent studies have uncovered significant evidence that the *Culicoides* biting midge (Diptera: Ceratopogonidae) could act as a vector of leishmaniasis caused by *Mundinia* species consisting of *L. martiniquensis*, *L. orientalis*, and *L.* sp. from Ghana [4]. 

Haemosporidian parasites, order Haemosporidia, infect a wide range of vertebrate species all over the world. These intracellular blood parasites are divided into three genera: *Leucocytozoon*, *Plasmodium*, and *Haemoproteus*. Insects primarily serve as their vectors, in which mosquitoes (Culicidae), black flies (Simuliidae), and biting midges (Ceratopogonidae) are vectors of *Plasmodium*, *Leucocytozoon*, and *Haemoproteus*, respectively [5]. There are roughly 1,400 *Culicoides* biting midge species that have been reported throughout the world [6]. Only a few species, however, act as disease vectors in both animals and humans. The biting midges, genus *Culicoides*, not only play a vital role in the transmission of avian *Haemosporidia* parasites, which can cause lethal avian malaria in non-adapted birds and domestic chickens but also of avian *Trypanosoma* [7]. *Culicoides* blood meal analyses help researchers understand more about the behaviour of these insects and the possibility of zoonotic disease transmission from animals to human beings. Earlier studies found that the *Culicoides* biting midges feed on a variety of hosts, including mammals and birds [8]. 

Due to the favourable climate for both sand fly and biting midge growth and development, the southern and northern parts of Thailand are now regarded as key endemic locations of autochthonous leishmaniasis [9]. Despite ongoing reports of leishmaniasis cases caused by *Mundinia* species in Thailand, the established vectors remain unknown [10]. Other studies from Thailand suggest that sand flies and biting midges are both potential vectors for autochthonous leishmaniasis and trypanosomiasis [9,11,12]. The study, conducted in Lamphun province, Northern Thailand, near the residence of a leishmaniasis patient, found *L. martiniquensis* and avian *Trypanosoma* DNA in collected biting midges, highlighting the role of *Culicoides* biting midges as potential vectors of both autochthonous leishmaniasis and trypanosomiasis [9].

The aim of the study is to investigate the *Leishmania* and *Trypanosoma* parasites in *Culicoides* biting midges collected from the affected area of autochthonous leishmaniasis in Phatthalung province, southern Thailand. Other pathogens that biting midges may act as possible vectors for, such as haemosporidians, were also tested. Additionally, in order to comprehend the interactions between vectors and hosts, the *Culicoides* host-feeding preferences were also investigated. 

## 2. Materials and Methods

### 2.1. Ethics Statement

The investigation protocol on animals was approved by the animal research ethics committee of Chulalongkorn University Animal Care and Use Protocol (CU-ACUP), Faculty of Medicine, Chulalongkorn University, Bangkok, Thailand (COA No. 019/2563).

### 2.2. Biting Midge Collection

**Figure 1:**
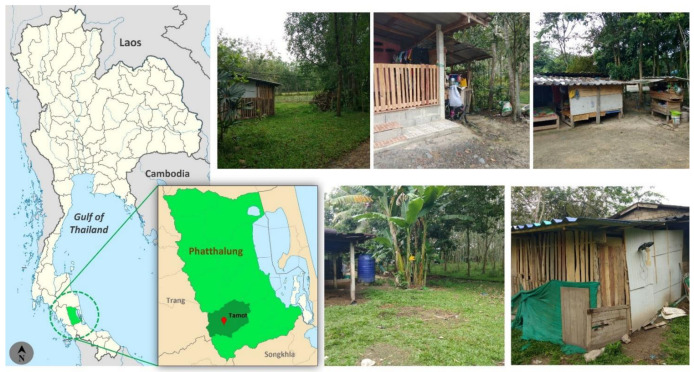
Map of Thailand (https://en.wikipedia.org/wiki/Phatthalung_province; accessed on 27 June 2022) and location of study, including the photographs of trap sites in Phatthalung province, southern Thailand. The photos were taken by Sakone Sunantaraporn, the first author.

Biting midges were trapped in the surroundings of the autochthonous leishmaniasis patient’s home. This study was conducted in Tamot District, Phatthalung Province of Southern Thailand (7°19′51.34″N 100°6′4.3″E) in October 2020. These flies were collected by using Center for Disease Control and Prevention miniature light traps with ultraviolet light (CDC-UV light). Four traps were placed 1.5 m above the ground around the patient’s home (Figure 1) and were continuously set up from 6.00 pm to 6.00 am for two nights. The collected insects in the bags were anesthetized by cooling on ice for 30 min. Subsequently, the female biting midges were morphologically separated from other insects under a stereomicroscope (Olympus, Tokyo, Japan).

Engorged and non-engorged female biting midges were sorted and placed individually in a 1.5 mL microcentrifuge tube containing lysis buffer and kept in an ice box before being sent to the Center of Excellence in Vector Biology and Vector Borne Diseases, Department of Parasitology, Faculty of Medicine, Chulalongkorn University. Engorged biting midges were used for the host blood meal identification, while non-engorged biting midges were screened for parasitic infections.

### 2.3. Morphological Identification of Biting Midge

Individual biting midges were dissected under a stereomicroscope with a single-use sterile needle. The head, wings, and genitalia were removed and mounted onto glass slides with Hoyer’s medium. Biting midge was identified according to morphological keys and descriptions by Wirth and Hubert [13]. The rest of the body parts were placed in a sterilized 1.5 mL microcentrifuge tube for DNA isolation.

### 2.4. DNA Isolation

Total DNA was isolated from the thorax and abdomen of individual biting midges. The flies were lysed in 100 µL of lysis buffer supplemented with 10 µL of proteinase K and grounded with a sterilized plastic pestle. DNA isolation was performed using an Invisorb^®^ Spin Tissue mini kit (STRATEC Molecular GmbH, Berlin, Germany) according to the manufacturer’s instructions. The DNA was eluted with 40 µL of elution buffer and then stored at −20 °C until use.

### 2.5. Molecular Identification of Host Blood Meals

**Figure 2:**
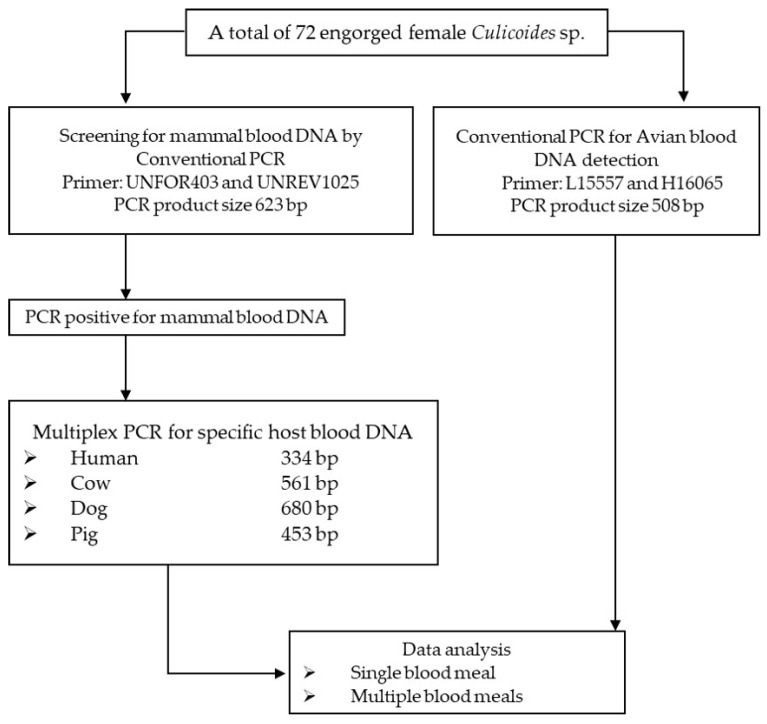
Experimental design for blood meal identification of female *Culicoides* biting midges from Phatthalung Province, Southern Thailand.

Isolated DNA from a total of 72 engorged female biting midges (of *Culicoides* species of *C. arakawae*, *C. fulvus*, *C. guttifer*, *C. gemellus*, *C. innoxius*, *C. insignipennis*, *C. jacobsoni*, *C. orientalis*, *C. peregrinus, C. shortti*, and *C.* subgenus *Trithecoides*) was used for the amplification of the mitochondrial cytochrome b (*cytb*) gene of the host DNA. An experimental design flow chart for blood meal identification of female *Culicoides* biting midges from Phatthalung Province, Southern Thailand is provided in Figure 2.

Primers UNREV1025 and UNFOR403 were used to screen for mammal DNA in all DNA samples [14]. The conventional PCR conditions for mammal blood screening were performed under the following conditions: denaturation at 95 °C for 5 min, then 35 cycles of 95 °C for 1 min, annealing at 58 °C for 1 min, and extension at 72 °C for 1 min, with a final extension at 72 °C for 7 min. Extracted DNA for human (*Homo sapiens*) blood was used as a positive control. Sterilized distilled water was used as a negative control. The positive sample for mammal DNA was subjected to a blood host confirmation process with primers specific to human (Human741F), cow (Cow121F), dog (Dog368F), and pig (Pig573F) by multiplex PCR [14]. The multiplex PCR conditions for blood hosts specific to humans, cows, dogs, and pigs are the same as for mammal blood screening. Extracted DNA from the blood of humans (*Homo sapiens*), cows (*Bos tarsus*), dogs (*Canis familiaris*), and pigs (*Sus scrofa*) was used as a positive control. Sterilized distilled water was used as a negative control. The remaining DNA from each specimen was further screened for avian blood.

For avian DNA identification, conventional PCR was performed with L15557 and H16065 primers in all samples as previously described [15]. The conventional PCR conditions for avian blood detection were as follows: initial denaturation at 95°C for 5 min, followed by 35 cycles of denaturation at 95°C for 1 min, annealing at 50°C for 1 min, extension at 72°C for 1 min, and a final extension after the last cycle at 72°C for 7 min. Extracted DNA from the chicken’s blood (*Gallus domesticus*) was used as a positive control. Sterilized distilled water was used as a negative control. All PCR amplification protocols were carried out by the Green Hot Start PCR master Mix Direct-Load 2X (Biotectrabbit, Berlin, Germany) according to the recommended instructions. The PCR amplicons were separated by 2% agarose gel electrophoresis, stained with ethidium bromide, and imaged with Quantity One quantification analysis software, version 4.5.2 Gel Doc EQ system (Bio-Rad, USA). All primer sequences and PCR conditions for the blood meal identification assay were provided in Table 1.

### 2.6. Molecular Detection of Leishmania, Trypanosoma, and Haemosporidian DNA

PCRs were carried out for parasite detection in 487 non-engorged female biting midges. Engorged female samples were not used in the experiment to avoid contamination by host blood. Primers specific for *Leishmania* and *Trypanosoma* were used to amplify the *ITS1* region (LeF 5′-TCCGCCCGAAAGTTCACCGATA-3′ and LeR 5′-CCAAGTCATCCATCGCGACACG-3′) [16] and *SSU rRNA* gene (TRY927F 5′-GAAACAAGAAACACGGGAG- 3′ and TRY927R 5′-CTACTGGGCAGCTTGGA-3′) [17], respectively. The PCR reagents and condition profiles were described by Srisuton et al. (2019) [12].

Nested PCR (nPCR) based on the mitochondrial *cytb* gene was performed for the detection of haemosporidian including *Plasmodium*, *Leucocytozoon*, and *Haemoproteus* [18]. PCR amplifications were undertaken in 25 µL volume, composed of 5 µL of template DNA, 25 mM of MgCl_2_, 1× PCR buffer, 2.5 mM of dNTPs, 10µM of each primer, and 1 unit of *Taq* DNA polymerase (Thermo Fisher Scientific, Walthman, MA, USA). The PCR conditions were as follows: initial denaturation at 94 °C for 3 min; 94 °C for 30 s; 53 °C for 30 s; and 72 °C for 45 s with the final extension at 72 °C for 10 min; and the second-round conditions were the same as the first-round PCR. The PCR reactions were performed for 20 and 35 cycles for the first and second rounds, respectively. PCR products were visualized by gel electrophoresis in a 1.5% agarose gel processed with Quantity One quantification analysis software, version 4.5.2 Gel Doc EQ system (Bio-Rad, Hercules, CA, USA). Positive and negative controls were included in all PCR amplification.

### 2.7. Sequence Analysis and Phylogenetic Tree Construction

All positive amplified products were ligated into the pGEM-T^®^ Easy Vector (Promega, Mandison, WI, USA) according to the manufacturer’s instructions. Plasmid DNA containing the suspected insert gene was extracted using the GeneAll^®^ Exprep^TM^ Plasmid purification kit (GeneAll Biotechnology, co., ltd, Seoul, Korea) following the manufacturer’s instructions. The purified plasmid DNA was submitted to the Macrogen Inc. (Seoul, South Korea) for sequencing service.

The obtained nucleotide sequences were manually edited to achieve the consensus sequences before alignment using ClustalW multiple alignments in BioEdit Sequence Alignment Editor Version 7.2.5 [19]. The consensus nucleotide sequences were then compared to the sequences available in the GenBank database using the nucleotide BLAST tool (https://blast.ncbi.nlm.nih.gov/Blast.cgi, accessed on 23 June 2022). The phylogenetic tree was generated by MEGAX software [20] based on the maximum likelihood method with 1000 bootstrap replicates. The generated *SSU rRNA* sequences were submitted to the GenBank database under the accession numbers OP217134 for *Trypanosoma* sp. and OP217135 and OP217136 for *Crithidia* species. The haemosporidian *cytb* sequences were deposited in GenBank (accession no. OP227012 for *P. juxtanucleare*; OP227013-OP227015 for *Leucocytozoon* sp.).

## 3. Results

### 3.1. Species Composition of Biting Midges

**Figure 3:**
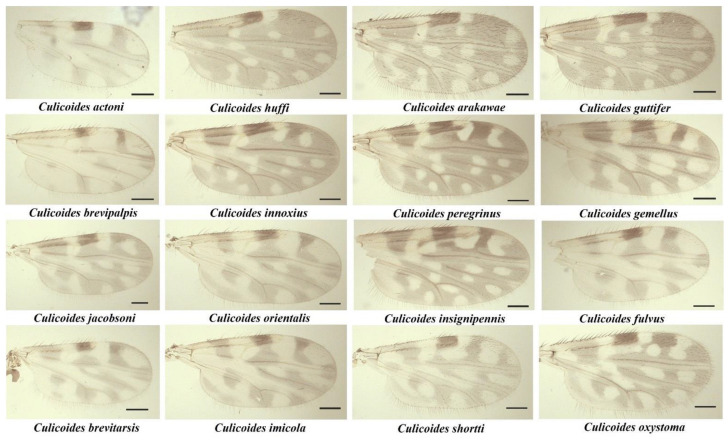
Photographs of wing patterns of 16 representatives *Culicoides* species collected from this study, scale bar: 100 µm.

A total of 559 female *Culicoides* biting midges were collected, belonging to 17 species from 6 subgenera, consisting of *Avaritia*, *Haemophoructus*, *Hoffmania, Meijerehelea, Remmia, Trithecoides,* and two groups of *Shortti* and *Clavipalpis* (Table 2). Of these, 72 (12.88%) were blood-engorged. Based on morphological identification, *C. peregrinus* (30.94%) was the most abundant *Culicoides* species found in the study area, followed by *C.* subgenus *Trithecoides* (24.87%). In addition, for the subgenus *Trithecoides*, the specimens could not be identified at the species level and, therefore, were identified by their morphologic characteristics, possessing a yellow scutum and three spermathecae. The wing patterns of the identified *Culicoides* species are shown in Figure 3.

### 3.2. Identification of Host Blood Meals

**Figure 4:**
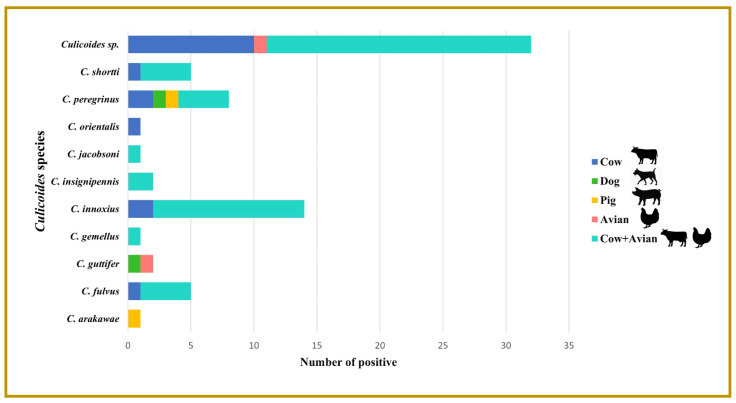
The frequency of host blood meals identified in *Culicoides* species collected from Phatthalung Province, Southern Thailand. Note: Human blood was not detected in specimens of engorged midges.

**Figure 5:**
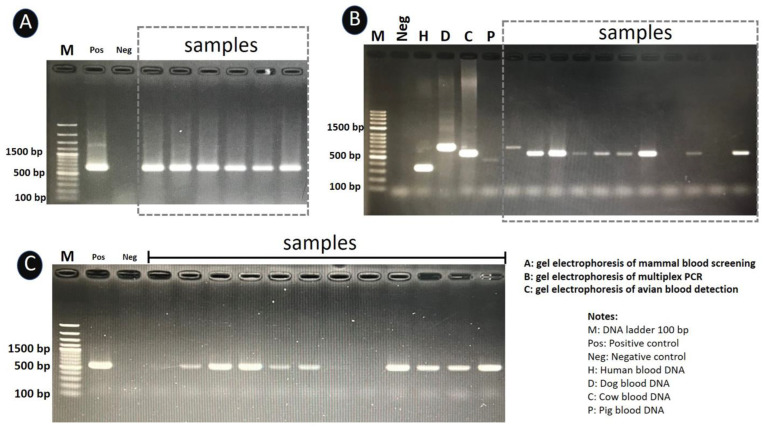
PCR amplification of the cytb gene against mammal blood DNA (**A**), human blood, pig blood, cow blood, dog blood DNA (**B**), and avian blood DNA (**C**) from Culicoides. Lane H, D, C, and P: human blood, dog blood, cow blood, and pig blood, respectively, lane M: DNA ladder (100 base pairs [bp]), lane Pos: positive control, lane Neg: negative control (no DNA template: Sterilized distilled water).

Identification of host blood was carried out on 72 specimens of blood-engorged *Culicoides* species of C. *arakawae*, *C. fulvus, C. guttifer*, *C. gemellus*, *C. innoxius, C. insignipennis*, *C. jacobsoni*, *C. orientalis*, *C. peregrinus*, *C. shortti*, and *C.* subgenus *Trithecoides*. Seventeen specimens revealed cow blood DNA (23.61%) in six species of *C.* subgenus *Trithecoides* (*n* =10), *C. peregrinus* (*n* =2), *C. innoxius* (*n* =2), *C. fulvus* (*n* =1), *C. orientalis* (*n* =1), and *C. shortti* (*n* =1). Dog blood DNA was detected in two (2.78%) specimens (one from *C. guttifer* and one from *C. peregrinus*). Furthermore, two (2.78%) specimens were positive for pig blood DNA (one from *C. arakawae* and one from *C. peregrinus*). Avian blood DNA was found in two specimens (2.78%) (one from *C. guttifer* and one from *C.* subgenus *Trithecoides*). The most prevalent blood meal pattern identified in this study was mixed host blood DNA (cow and avian) standing at 49 (68.05%) (*C.* subgenus *Trithecoides* (*n* =21), *C. innoxius* (*n* =12), *C. peregrinus* (*n* =4), *C. shortti* (*n* =4), *C. fulvus* (*n* =4), *C. insignipennis* (*n* =2), *C. jacobsoni* (*n* =1), and *C. gemellus* (*n* =1)) (Figure 4). The photographs of the PCR gel results are provided in Figure 5.

### 3.3. Detection of Leishmania, Trypanosoma, and Haemosporidian DNA

**Figure 6:**
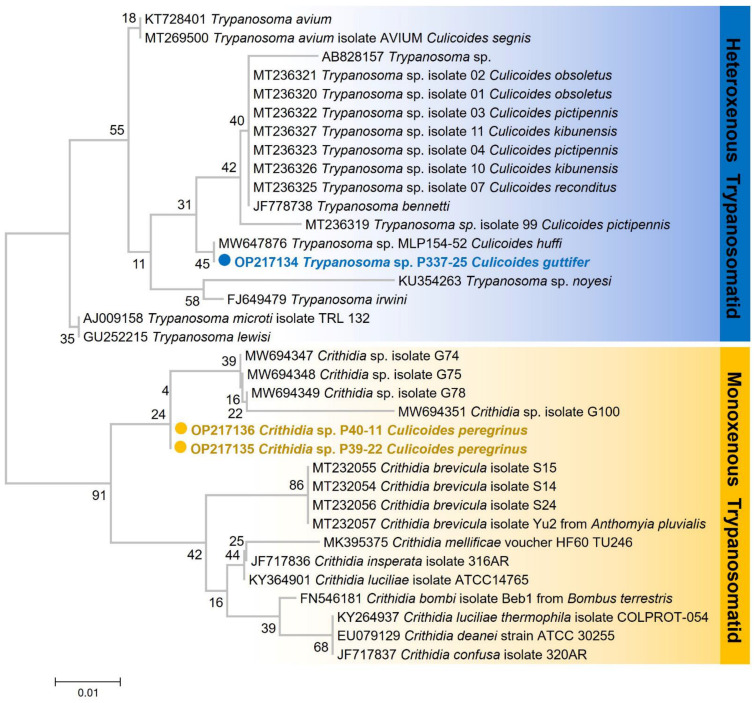
Phylogenetic tree based on partial *SSU rRNA* sequences of Trypanosomatids including *Trypanosoma* and *Crithidia* parasites. The tree was constructed using the maximum likelihood method with the K2+G model, generated by the 1000 bootstrap tests.

A total of 487 non-engorged biting midges were screened for *Leishmania*, *Trypanosoma*, and haemosporidian parasites (Table 2). We detected *Trypanosoma* sp. in one specimen of *C. guttifer*. The nucleotide BLAST result of this parasite was compared with those sequences available in the GenBank database, demonstrating a high similarity of 99.79% with *Trypanosoma* sp. (accession no. MW647876) found in *C. huffi* previously reported in Northern Thailand. Additionally, two unknown *Crithidia* sp. were also detected in *C. peregrinus* by using TRY927F and TRY927R primers that were specific to the *SSU rRNA* gene. Sequence comparison of the two *Crithidia* sp. with data available in the GenBank showed 98.92 % and 99.03 % similarities with sequences of *Crithidia* found in *Glossina fusipes fusipes* (accession no. MW694347). The maximum likelihood tree demonstrated that *Trypanosoma* sp. detected in the study was classified as avian Trypanosomes while the *Crithidia* sp. was grouped within conspecific species (Figure 6). No *Leishmania* DNA was detected in the collected biting midges of this study.

For haemosporidian detection, nPCR based on the haemosporidian *cytb* gene was positively detected in four (0.82%) specimens. *Leucocytozoon* sp. was found in three (0.62%) specimens, one from each species: *C. fulvus*, *C. oxystoma*, and *C.* subgenus *Trithecoides*. Moreover, *Plasmodium* species was detected in one (0.20%) specimen from C. guttifer (Table 2). Comparisons of the obtained *cytb* sequences of *Plasmodium* sp. with sequences published in the GenBank database demonstrated that the sequence was 99.43% compatible with *P.* *juxtanucleare* (accession no. KU248847) found in *Gullus gullus spadiceus*. Additionally, the BLASTn results of all *Leucocytozoon* revealed 99.00–99.20% similarities with an unidentified *Leucocytozoon* sp. (accession no. KT290940) reported in chicken (*Gullus gullus spadiceus*) from Thailand. 

**Figure 7:**
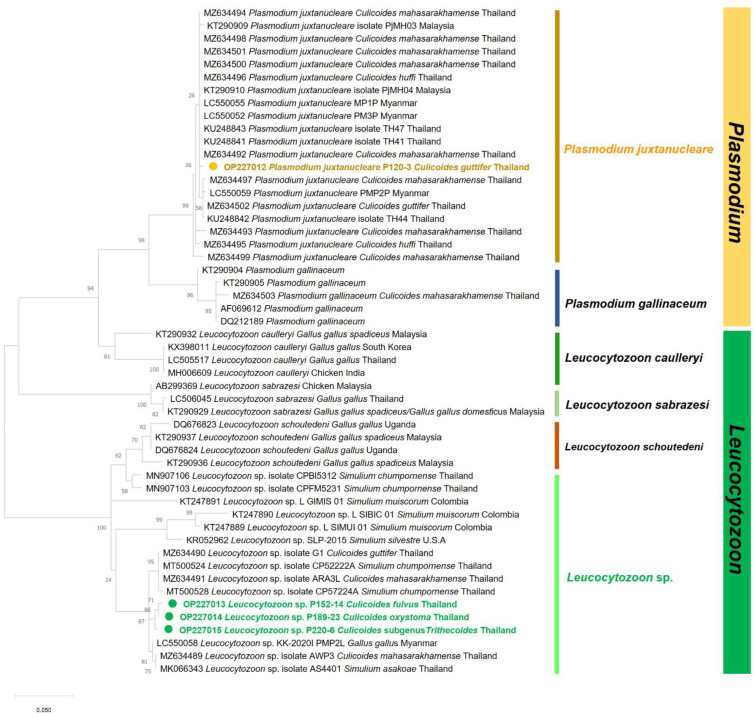
Maximum likelihood method (ML) tree of haemosporidian *cytb* sequences created by the K2+I model with 1000 bootstrap tests.

The ML tree demonstrated that the *P. juxtanucleare* detected in *C. guttifer* from our study belonged to a clade of *P. juxtanucleare* with reports of infecting avian hosts and several *Culicoides* species from different geographic regions. Moreover, the tree revealed that *Leucocytozoon* sp. detected in *C. fulvus*, *C. oxystoma*, and *C.* subgenus *Trithecoides* were genetically related to *Leucocytozoon* sp. found in domestic chickens (*Gallus gallus*), black flies (*Simulium chumpornense* and *S. asakoae*), and biting midges (*C. mahasarakramense* and *C. guttifer*) reported in Thailand (Figure 7).

## 4. Discussion

Our study assessed the role of *Culicoides* biting midges as a potential vector of emerging zoonotic diseases and determined the host range of biting midges collected from leishmaniasis-endemic areas. The study uncovered an intriguing finding of a range of pathogens carried by midges collected in Southern Thailand. Some of the findings supported the relatively new hypothesis that zoonotic diseases in Thailand may be transmitted by biting midges [21]. Among all the specimens that were gathered, *C. peregrinus* was by far the most abundant species of *Culicoides*. The majority of blood-fed *Culicoides* samples displayed a pattern of mixed blood DNA from cow and avian hosts. The overall field infectivity rate of the non-engorged midges was 1.4%, with the detection of *Leucocytozoon* sp. and *Crithidia* sp. separately in three and two *Culicoides*, respectively. *Trypanosoma* sp. and *P. juxtanucleare* were also detected individually in two *C. guttifer* specimens.

Blood meal analysis in our study found that most biting midges fed on two hosts rather than one, with blood meals from cows and avians being the most prevalent feeding preferences (68%). Biting midges also fed separately on cows, avians, dogs, and pigs. Disregarding mixed-blood feeding patterns, most of the blood ingested by engorged biting midges came from cows. A recent study in Romania found mixed-blood meals from a bird and a mammal and two different mammal hosts in the same *Culicodes*. This occurred in approximately 0.8% of all specimens, which is lower than in our study [8]. Our results contradict most previous studies, which indicate that *Culicoides* are either mammalophilic or ornithophilic and feed primarily on a single blood source [8,22,23]. Blood source identification in engorged vectors helps comprehend vector–host interactions and pathogen ecology [24,25]. In Europe, the most identified vertebrate hosts of midges are ruminants, which is similar to our results [8,22,26,27,28]. Other animals, including humans, pigs, and, less frequently, avians, have been observed to serve as hosts for engorged midges [22,23,29,30,31,32]. Previous reports of blood meal analysis in *Culicoides* in Thailand yielded different conclusions from ours in that the majority of midge hosts were chickens, followed by cattle [33].

Regarding the blood-feeding preferences of biting midges, different species of *Culicoides* may prefer different hosts, and the spectrum of the predominant host in the area where the specimen was collected may also play a part [8,34]. In terms of biting midge dynamic feeding patterns in relation to host availability, it has been observed that the blood source for biting midges is associated with host availability near trap locations. The traps of this study were located in the surroundings of the reported leishmaniasis patient, which was in a rural area where livestock farming was one of the main income sources. Due to the proximity of the trap to dairy and poultry farms, cows and avians are the most readily available hosts for biting midges collected in this study. This led to the use of the primers targeting the mentioned animal blood, prioritizing the use of mammal primers. If mammal blood was detected, it would support the hypothesis that *Culicoides* may serve as a vector for humans, transmitting the parasite from mammals to humans. The observed animals in the area, in conjunction with the host preferences of the biting midges, may explain why cows and avians are the most prevalent hosts in blood meal analysis. It has been discovered that *Culicoides* species feed on both avians and mammals. Nevertheless, they favour one host over the other. Our analysis revealed the high frequency of engorged midges feeding on cows and avians in a single individual, which may have been influenced by several factors. Changes in the host-seeking behaviour of biting midges may result from the trap area’s dynamic environment. Every nightfall at the patient’s farm, the patient’s family members will cover the traditional bamboo chicken coop with sheets and light a bonfire nearby to protect the farm animals from insects. This occurrence could limit the accessibility of avian hosts compared to cows. Since *Culicoides* can feed on both mammals and avians, they might switch to the other available host whenever their feedings are interrupted in order to obtain sufficient blood to produce eggs. We hypothesize that ornithophilic midges may be interrupted while feeding by environmental influences, as described previously, and must therefore switch hosts during that feeding. In this case, another accessible host may be a cow, as the patient’s dairy and poultry farms were not completely isolated and were located close to one another. This assumption might be the explanation for the high number of mixed-blood meals in our study. Even though there have been reports of midges with host preferences, the vast majority of *Culicoides* species have no host specificity and feed on a wide range of hosts. Therefore, they may serve as significant potential bridge vectors. The blood analysis of the *Culicoides* collected during this study did not reveal any evidence of feeding on human blood. However, the possibility that biting midges might be a vector of an unidentified zoonotic disease in humans remains to be discovered. The other potential factor contributing to the high number of mixed-blood meal results could be the study’s approach.

In this study, we adapted the methods of a mammalian-specific multiplexed primer set based on cytochrome b that Kent et al. [14] used to detect the blood hosts of engorged mosquitoes for the purpose of detecting the blood hosts of midges. This PCR diagnostic technique recognizes mammalian blood hosts by size-specific fragments after agarose gel electrophoresis. Kent et al. developed this diagnostic test to identify the blood meals of malaria-transmitting mosquitoes captured in African villages, where humans and domestic animals are the most likely hosts [14]. This test is applicable in any setting where potential blood hosts such as humans, cattle, dogs, goats, and pigs are present. Later, Siriyasatien et al. [35] and Boonserm et al. [36] employed this method to determine the blood meals of field-caught mosquitoes in Thailand. Interestingly, *Leishmania* DNA was identified in biting midges in a recent investigation conducted in Lamphun province, northern Thailand, near the residence of a leishmaniasis patient [9]. Therefore, there is a rising effort to discover whether or not these insects are the potential vectors of autochthonous leishmaniasis in Thailand. This study intends to determine whether midges surrounding the leishmaniasis patient’s residence exhibit evidence of feeding on humans. We selected this method due to its emphasis on mammalian hosts’ blood detection, as humans are the primary species of interest in our study. In addition, this approach has high sensitivity and specificity and is also cost-saving. However, this is a relatively new method for detecting *Culicoides*’ host blood meals. More research is required to compare the sensitivity and specificity of this method to the more frequently used method, sequence-based analysis. In addition, a limitation of relying solely on PCR is that we cannot determine the host at the species level. The potential methodological biases imposed by the study design should, therefore, not be disregarded. We chose to identify only mammalian and avian hosts for our blood meal study, thus eliminating the chance of discovering other vertebrate hosts. In addition, we trapped midges in a single location, so the sample size of midges subjected to blood meal analysis was quite limited. As a result, it did not adequately reflect the preferences of midge hosts in Thailand.

In addition to sand flies, biting midges of the genus *Culicoides* were found as a potential vector of autochthonous leishmaniasis in Thailand for the first time in 2021 [9]. Sunantaraporn et al. [9] found that all of the identified *L. martiniquensis* were detected in *C. mahasarakhamense*. However, we were unable to detect *Leishmania* DNA in the *Culicoides* collected from the leishmaniasis-endemic district of Phatthalung in Thailand. The inability to detect the *Leishmania* parasite in the collected biting midges in our study could be attributed to a smaller number of the favourable *Culicoides* species of *Leishmania*, likely *C. mahasarakhamense*, than in the previous study. Other possible factors include seasonality at the time of collection, which may not be conducive to disease propagation among insects, the absence of a reservoir host in that location, the severity of the disease outbreak during collection, and the susceptibility of vectors in a specific area [37,38]. Four species of parasites belonging to two parasitic groups, haemosporidian (*P. juxtanucleare* and *Leucocytozoon* sp.) and trypanosomatid (*Crithidia* sp. and *Trypanosoma* sp.) parasites, were identified in *Culicoides* biting midges collected for this study.

Haemosporidian infections in domestic hens and wild birds have been expanding in Thailand [39,40,41,42]. In the study by Pramual et al., [21] it was demonstrated that *C. mahasarakhamense*, *C. guttifer*, and *C. huffi* were infected with *Leucocytozoon* and two of *P*. *juxtanucleare* and *P*. *gallinaceum*, making them potential vectors for these parasites. Additionally, in our investigation, we also detected *Leucocytozoon* sp. in *C. fulvus*, *C. oxystoma*, and *C.* subgenus *Trithecoides*, and *P. juxtanucleare* in *C. guttifer*, which adds support to the hypothesis that biting midges could potentially transmit more forms of haemosporidian than previously known.

Trypanosomatids are divided into two groups: heteroxenous (involving vertebrates and insect vectors) and monoxenous (lower trypanosomatids), which are thought to be unique parasites of invertebrate hosts [43]. In this study, we detected both heteroxenous (*Trypanosoma* sp.) and monoxenous (*Crithridia* sp.) trypanosomatids in biting midges from a southern Thai province. The identified heteroxenous trypanosomatid shared 99.79 percent similarity with avian *Trypanosoma* species previously reported in Northern Thailand [9]. While the field infection rate of *Trypanosoma* sp. in *C. huffi* from a previous study was 4.76% (1/21), ours was slightly lower at 2.5% (1/40). Our results support the findings by Sunantaraporn et al. that, in addition to tsetse flies, which are the primary vectors of trypanosome parasites that cause trypanosomiasis [44], *Culicoides* biting midges may also be possible vectors of avian trypanosomiasis.

The majority of insect monoxenous trypanosomatids are regarded as non-pathogenic or even commensals, but they can occasionally infect humans and other mammals [45,46,47]. Important findings contradict the assumption that lower trypanosomes are monoxenous and exclusive to insects. Moreover, their host specificity is complex and poorly understood [43,48]. Two *Crithidia* sp. sequences identified in our study were closely related to the species found in tsetse flies in the study by Votypka et al. [45]. *Crithidia* sp. are insect gut parasites. Their common insect hosts include *Bombus hortorum*, *B. muscorum*, *B. terrestris*, and *Culex* spp. [49]. Although it is believed that the infection in an insect was acquired directly through the host’s blood, a recent study by Votypka et al. [45] explored the possibility of alternative explanations that insects become infected after consuming contaminated sugary meals and water. However, due to the fact that midges exhibit a variety of host blood-sucking behaviours and cohabitation has been documented, for the detection of infection in this study, we focused primarily on direct exposure to the host’s blood. Several studies have demonstrated the occurrence of lower trypanosomatids, which include *Crithidia* species, in immunocompromised patients, and it was recently discovered that they can cause leishmania-like symptoms and co-infections with *Leishmania* in immunocompetent individuals [43,50,51,52,53]. *Crithidia* spp. closely related to the *C. fasciculata* species were identified in patients initially suspected of having cutaneous leishmaniasis in Iran [50]. In Thailand, the incidence of difficult-to-treat autochthonous leishmaniasis is rising. Given the possibility of *Crithidia* causing a clinical presentation resembling that of leishmania infection, a misdiagnosis or co-infection with *Crithidia* may be the cause. Attention should be focused on the natural hosts and animal reservoirs of these pathogens. The ecology of *Crithidia* that contributes to the emerging diseases in humans should be further investigated.

Based on the results of our study, we hypothesized that midges could potentially transmit specific types of haemosporidian and trypanosomatid parasites to other animal hosts and cause zoonotic diseases in susceptible animal species. The experimental proof is necessary to determine whether biting midges are competent vectors for the aforementioned parasites, which are defined by their capacity to acquire a disease, sustain its reproduction, disperse it, and transmit it to other organisms [54,55].

## 5. Conclusions

Our evidence indicates that biting midges have diverse feeding preferences and might be capable of transmitting a variety of pathogens, including avian haemosporidian and trypanosomatid parasites. In our study, however, we did not compare morphological aspects with genetic characterization using molecular approaches in midges. This could prevent the discovery of new species of *Culicoides* biting midges. In addition, because the sample size of the engorged biting midges may be insufficient because of the short trapping period in a single location, the interpretation of the blood analysis may not precisely reflect the actual condition. Ultimately, it cannot be inferred that biting midges are natural vectors based merely on the detection of parasite DNA, and additional research covering more sample sizes and regions is required. Additionally, emerging diseases caused by *Culicoides* biting midges might still be underappreciated or misdiagnosed. To mitigate this consequence, we may need to focus more on biting midge ecology and surveillance research in the future in order to better understand the current situation and gain firmer control over biting midge populations.

## Figures and Tables

**Table 1:** Primer sequences and PCR conditions for the cytochrome b-based polymerase chain reaction blood meal identification assay.

Primer	Sequence (5′-3′)	PCR Conditions	Target Host	Product Size (bp)	References
**Conventional PCR condition for mammal blood screening**					
**UNFOR403**	TGAGGACAAATATCATTCTGAGG	95 °C, 5 m; 35 cycles of 95 °C 1 m, 58 °C 1 m, 72 °C 1 m; 72 °C 7 m	Mammals	623	[14]
**UNREV1025**	GGTTGTCCTCCAATTCATGTTA				[14]
**Multiplex PCR conditions for blood host specific for humans, cows, dogs, and pigs**					
**Human741F**	GGCTTACTTCTCTTCATTCTCTCCT	95 °C, 5 m; 35 cycles of 95 °C 1 m, 58 °C 1 m, 72 °C 1 m; 72 °C 7 m	Human	334	[14]
**Cow121F**	CATCGGCACAAATTTAGTCG		Cow	561	[14]
**Dog368F**	GGAATTGTACTATTATTCGCAACCAT		Dog	680	[14]
**Pig573F**	CCTCGCAGCCGTACATCTC		Pig	453	[14]
**UNREV1025**	GGTTGTCCTCCAATTCATGTTA			-	[14]
**Conventional PCR condition for avian blood detection**					
**L15557**	GACTGTGACAAAATCCC[A/G/C/T]TTCCA	95 °C, 5 m; 35 cycles of 95 °C 1 m, 50 °C 1 m, 72 °C 1 m; 72 °C 7 m	Avian	508	[15]
**H16065**	GGTCTTCATCT[C/T][A/T/C]GG[C/T]TTACAAGAC			-	[15]

**Table 2:** Species Abundance, Number of Blood Fed, and Parasitic Detection Collected in Thai *Culicoides* biting midges, from Phatthalung province, southern Thailand.

Subgenus	Species	No.	Blood Fed (n)	Non-Engorged Specimens (*n* = 487) ^a^	
				**Trypanosomatids (n)**	**Haemosporidians (n)**
*Avaritia*	*C. actoni*	1	0	-	-
*Avaritia*	*C. brevipalpis*	2	0	-	-
*Avaritia*	*C. imicola*	2	0	-	-
*Avaritia*	*C. jacobsoni*	2	1	-	-
*Avaritia*	*C. brevitarsis*	4	0	-	-
*Avaritia*	*C. orientalis*	9	1	-	-
*Avaritia*	*C. fulvus*	33	5	-	*Leucocytozoon* sp. (1)
*Haemophoructus*	*C. gemellus*	13	1	-	-
*Hoffmania*	*C. innoxius*	38	14	-	-
*Hoffmania*	*C. peregrinus*	173	8	*Crithidia* sp. (2)	-
*Hoffmania*	*C. insignipennis*	4	2	-	-
*Meijerehelea*	*C. arakawae*	34	1	-	-
*Meijerehelea*	*C. guttifer*	42	2	*Trypanosoma* sp. (1)	*P. juxtanucleare* (1)
*Remmia*	*C. oxystoma*	5	0	-	*Leucocytozoon* sp. (1)
*Shortti* group	*C. shortti*	37	5	-	-
*Clavipalpis* group	*C. huffi*	21	0	-	-
*Trithecoides*	*Culicoides* sp.	139	32	-	*Leucocytozoon* sp. (1)
**Total**		**559**	**72**	**3**	**4**

^a^ Specimens used for the detection of pathogens.

## Data Availability

All relevant data are within the manuscript.
